# *In Vitro* and *in Vivo* Evaluation of Lactoferrin-Conjugated Liposomes as a Novel Carrier to Improve the Brain Delivery

**DOI:** 10.3390/ijms14022862

**Published:** 2013-01-29

**Authors:** Feng-Yun J. Huang, Wan-Jou Chen, Wan-Yu Lee, Su-Tang Lo, Te-Wei Lee, Jem-Mau Lo

**Affiliations:** 1 Department of Biomedical Engineering and Environmental Sciences, National Tsing Hua University, Hsinchu 30013, Taiwan; E-Mails: jimmyhuang1007@gmail.com (F.-Y.J.H.); d948503@oz.nthu.edu.tw (W.-J.C.); wyfishlee@gmail.com (W.-Y.L.); 2 Department of Radiology, University of Texas Southwestern Medical Center, Dallas, Texas 75390, USA; E-Mail: Su-Tang.Lo@utsouthwestern.edu; 3 Institute of Nuclear Energy Research, Longtan 32546, Taiwan; E-Mail: twlee@iner.gov.tw

**Keywords:** lactoferrin, PEGylated liposome, blood-brain barrier, ^99m^Tc

## Abstract

In this study, lactoferrin-conjugated PEGylated liposomes (PL), a potential drug carrier for brain delivery, was loaded with radioisotope complex, ^99m^Tc labeled *N*,*N*-bis(2-mercaptoethyl)-*N*′,*N*′-diethylethylenediamine (^99m^Tc-BMEDA) for *in vitro* and *in vivo* evaluations. The hydrophilicity of liposomes was enhanced by PEGylation which was not an ideal brain delivery system for crossing the blood brain barrier (BBB). With the modification of a brain-targeting ligand, lactoferrin (Lf), the PEGylated liposome (PL) might become a potential brain delivery vehicle. In order to test the hypothesis *in vitro* and *in vivo*, ^99m^Tc-BMEDA was loaded into the liposomes as a reporter with or without Lf-conjugation. The mouse brain endothelia cell line, bEnd.3 cells, was cultured to investigate the potential uptake of liposomes *in vitro*. The *in vivo* uptake by the mouse brain of the liposomes was detected by tissue biodistribution study. The results indicated that Lf-conjugated PEGylated liposome showed more than three times better uptake efficiency *in vitro* and two-fold higher of brain uptake *in vivo* than PEGlyated liposome. With the success of loading the potential Single Photon Emission Tomography (SPECT) imaging probe, ^99m^Tc-BMEDA, Lf-PL might serve as a promising brain delivery system for loading diagnostics or therapeutics of various brain disorders.

## 1. Introduction

The blood-brain barrier (BBB) is a unique structure which is formed by the brain endothelial cells lined with cerebral capillaries, together closely with perivascular neurons, astrocytic end-foot, and pericytes [1]. The main role of the BBB is to protect the central nervous system from exogenous toxicants, but on the other aspect, this structure is the most redoubtable obstacle for drug delivery for the treatment of brain disorders [2]. One prospective modality for delivering drugs for brain tumor treatment and for transporting the nuclear imaging probe for neurodegenerative diseases diagnosis are via the receptor-mediated targeting on luminal side of the BBB [3]. Lactoferrin (Lf, MW ~80 kDa), a single-chain iron-binding glycoprotein containing 690 amino acids folded into two globular lobes, is part of transferrin (Tf) family [4] which can penetrate the BBB via receptor-mediated transcytosis [5]. Due to the less concentration of endogenous Lf than Tf, Lf was observed to exhibit better BBB uptake than Tf resulting by the less competitive inhibition [6]. As such, Lf has been used to enhance the polymer-based drug delivery system and superparamagnetic iron oxide (SPIO)-based MRI contrast agent to brain [7–9]. Study further showed the applicability of using Lf-conjugated polymersome as drug carrier for the treatment of glioma in rodent model [10]. Moreover, the lactoferrin receptor density had been found increase in the mesencephalon region of patient with Parkinson disease [11]. Studies also showed the accumulation of lactoferrin in the lesions from different neurodegenerative diseases such as Alzheimer’s disease, Down syndrome, and Pick’s disease [12,13].

Liposomes, a cell membrane-like phospholipid-bilayer structure, contain a hydrophilic phase inside the core and a lipophilic phase between the bilayers. The biocompatible liposome nanoparticles can be loaded with hydrophilic drugs in the internal water compartment and hydrophobic drugs into the membrane [14]. The size, charge and surface properties of the liposome nanoparticles can be easily changed or modified by adding new ingredients to the phospholipid mixture during fabrication. Most recently, Chen *et al.* [15,16] developed Lf-modified procationic liposomes as a drug carrier for brain delivery. In this study, we used the radioisotope ^99m^Tc as a reporter of the PEGylated liposomes (PL) by loading ^99m^Tc-*N,N*-bis(2-mercaptoethyl)-*N*′,*N*′-diethylethylenediamine (^99m^Tc-BMEDA) into the liposome core. Cell-based *in vitro* brain delivery efficacy and *in vivo* tissue biodistribution of the PEGylated liposomes with or without Lf-conjugation will be discussed in detail.

## 2. Results and Discussion

### 2.1. Conjugation of Lactoferrin (Lf) to PEGylated liposome (PL)

**Figure 1:**
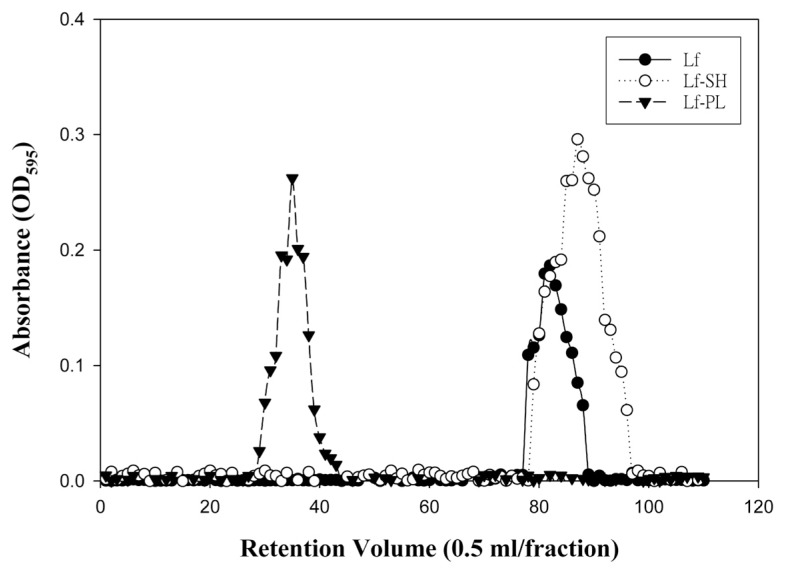
Chromatograms of Lf-PL together with Lf and Lf-SH on a Sepharose^TM^ 4B gel-filtration column eluted with normal saline.

The neutral PEGylated liposomes (PL) was fabricated with zeta potential as −0.60 ± 0.51 mV and particle size as 91.23 ± 17.88 nm with polydispersity index (PDI) of 0.12 ± 0.02 (*n* = 3). The thiolated Lf (Lf-SH) was validated by Ellman’s assay with the content of the terminal sulfhydryl group (-SH) on Lf-SH at 0.0243 ± 0.001 mM (*n* = 3). The Lf-PL was purified by a Sepharose^TM^ 4B gel-filtration column eluted with normal saline with the chromatogram as shown in Figure 1. The zeta potential of the final product of Lf-PL was slightly negative as −25.4 mV and its particle size measured to be 96.68 ± 17.84 nm with PDI of 0.14 ± 0.03 (*n* = 3).

The particle size is an important factor that affects the liposome endocytosis in the brain capillary cells. In our study, the size of the prepared PL[^99m^Tc] and Lf-PL[^99m^Tc] were between 75 and 120 nm, indicating a favorable condition for brain drug delivery by liposomes [17,18]. The particle size of Lf-PL[^99m^Tc] slightly increased about 5 nm in comparison with that of PL[^99m^Tc].

### 2.2. Characterization of PL[^99m^Tc] and Lf-PL[^99m^Tc]

The original concentration of phospholipid in the prepared PL solution was 16.7 ± 0.01 μmole/mL and the concentration of phospholipid in the final working Lf-PL solution was determined to be 4.17 ± 0.013 μmole/mL. From Bradford assay, the concentration of Lf in the Lf-PL solution was found to be 0.238 ± 0.0058 mg/mL. Based on these values, the number of Lf molecule in each Lf-PL particle was calculated to be 63.3 ± 2.17. Accordingly, the coupling efficiency of Lf to Lf-PL was estimated to be 74%.

The achievable radiochemical yield of ^99m^Tc-BMEDA was greater than 98%. For loading of the radioisotope complex, ^99m^Tc-BMEDA, into PL and Lf-PL liposomes, the loading yield of PL[^99m^Tc] (75%) was better than Lf-PL[^99m^Tc] (26%) that might be due to the steric obstruction of the additional Lf ligand. After purification by PD-10 column, the radiochemical purity of either PL[^99m^Tc] or Lf-PL[^99m^Tc] was around 96%.

### 2.3. Stability Study

**Figure 2:**
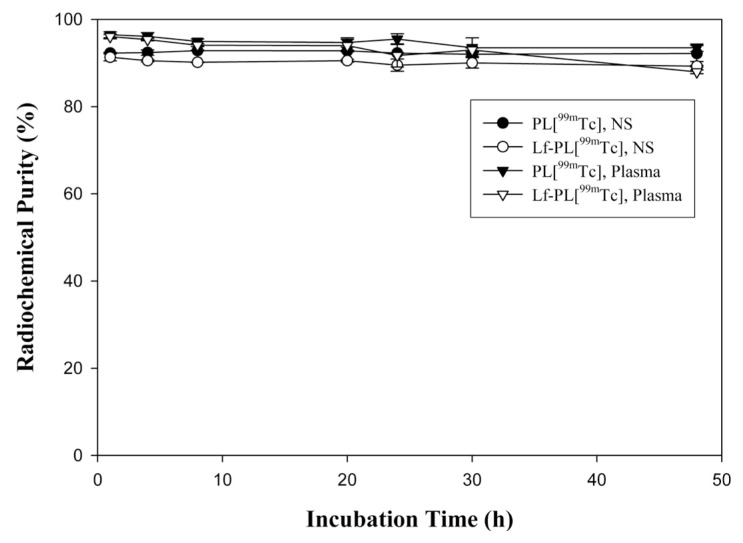
Stability of PL[^99m^Tc] and Lf-PL[^99m^Tc] during incubation in normal saline at room temperature and rat plasma at 37 °C (mean ± SD, *n* = 3).

The stability of PL[^99m^Tc] and Lf-PL[^99m^Tc] during incubation in normal saline at room temperature and rat plasma at 37 °C is shown in Figure 2. Either PL[^99m^Tc] or Lf-PL[^99m^Tc] showed high stability with over 87% intact after 48 h of incubation in both conditions which was suitable for *in vitro* and *in vivo* studies.

### 2.4. *In Vitro* Cell Uptake Study for Evaluation of BBB Penetration Potential

Current most common methods for modeling the BBB penetration include *in situ* perfusion model in animal and *in vitro* culture of endothelial cells. In this study, we used the rat brain endothelia cells, bEnd.3, to mimic the endogenous microvascular endothelial cells due to its expression of tight junction proteins which had been reported to a suitable BBB model for *in vitro* brain delivery study [19].

**Figure 3:**
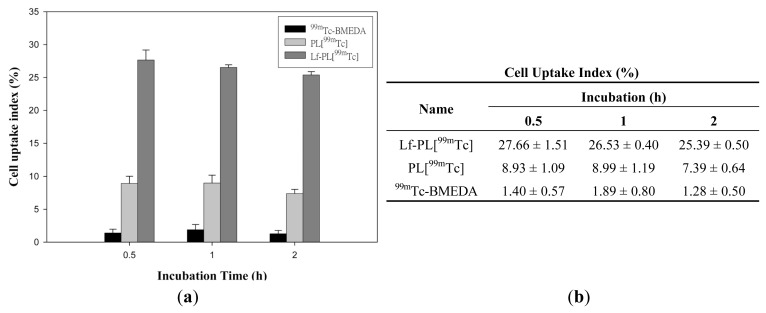
*In vitro* bEnd.3 cell uptakes of ^99m^Tc-BMEDA, PL[^99m^Tc], and Lf-PL[^99m^Tc] during incubation at 37 °C (mean ± SD, *n* = 3).

The *in vitro* cell uptake index of Lf-PL[^99m^Tc], PL[^99m^Tc], and ^99m^Tc-BMEDA was evaluated in a mouse brain endothelia cell line, bENd.3 cells, with incubation at 37 °C for 0.5, 1, and 2 h (Figure 3). The incubation time did not affect the uptake level in all three groups. However, Lf-PL[^99m^Tc] showed significant higher uptake compared to PL[^99m^Tc] or ^99m^Tc-BMEDA. The significant higher uptake values (*p* < 0.0001) between Lf-PL[^99m^Tc] and PL[^99m^Tc] in bEnd.3 cells indicated that the enhanced uptake efficacy was mediated by Lf receptor. This receptor was also found high level of expression in brain endothelial capillary cells (BCECs) [20]. Hence, further examination of *in vivo* animal study with Lf-conjugated liposome is needed to validate more precisely the relationship with affinity between Lf and Lf receptor in the BBB.

### 2.5. Pharmacokinetic Study

**Figure 4:**
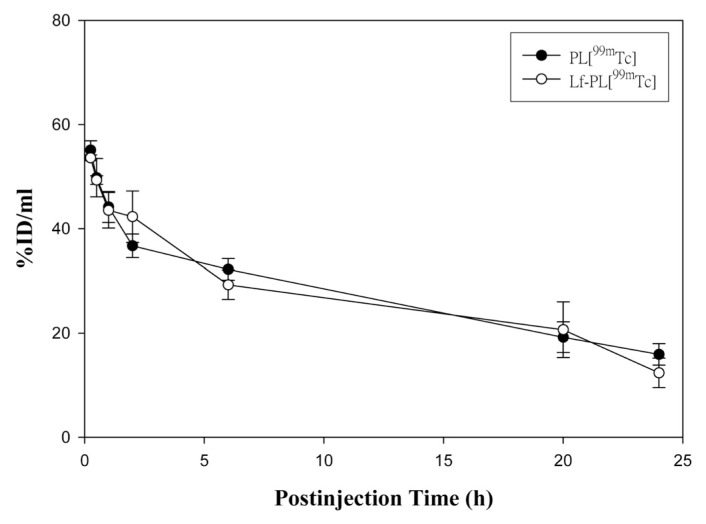
The clearance curves of PL[^99m^Tc] (●), and Lf-PL[^99m^Tc] (○) from blood (mean ± SD, *n* = 3).

The pharmacokinetic parameters of the clearance curves of PL[^99m^Tc] and Lf-PL[^99m^Tc] (Figure 4) are summarized in Table 1. Both of the values of the area under the curve (AUC_0→24h_) and the clearance rate (Cl) from Lf-PL[^99m^Tc] showed no significant difference to PL[^99m^Tc] with *p*-values of 0.89 and 0.31, respectively. From this pharmacokinetic examination, the Lf-conjugated liposomes can provide the similar long-circulation property *in vivo* as well as Lf-unconjugated liposomes and beneficially improve the drug delivery. For designing a better Lf-PL[^99m^Tc] to target Lf receptor, the number of Lf ligand on the liposomes should have a suitable level. Herewith, we loaded ~63 of Lf ligand on the liposome in this study which was similar to the previous report [8].

### 2.6. Biodistribution Study

**Figure 5:**
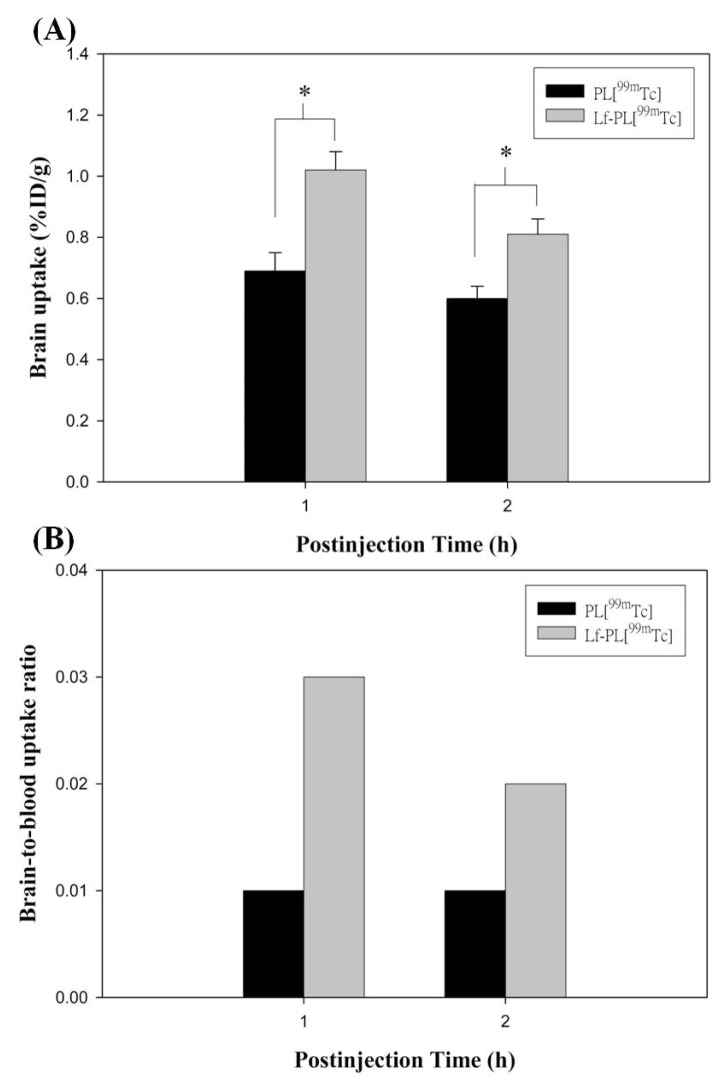
(**A**) Brain uptakes of PL[^99m^Tc] and Lf-PL[^99m^Tc] in BALB/c mice at 1 and 2 h post-injection. Note: (mean ± SD, *n* = 3), * indicates *p* < 0.05. (**B**) Brain-to-blood uptake ratios for PL[^99m^Tc] and Lf-PL[^99m^Tc] in BALB/c mice at 1 and 2 h post-injection.

The biodistribution results for PL[^99m^Tc] and Lf-PL[^99m^Tc] in BALB/c mice for 1 and 2 h post-injection are summarized in Table 2 and Figure 5. The brain uptake of Lf-PL[^99m^Tc] presented 1.47 ± 0.16 fold and 1.34 ± 0.12 fold higher than PL[^99m^Tc] at 1 and 2 h postinjecton, respectively (*p* < 0.05) (Figure 5A). The brain-to-blood ratios of Lf-PL[^99m^Tc] were 3-fold and 2-fold higher than PL[^99m^Tc] for 1 and 2 h post-injection, respectively (Figure 5B). It was noteworthy that the accumulation of Lf-PL[^99m^Tc] in the spleen was obviously higher than that of PL[^99m^Tc], with the uptake values presented as 34.12% ± 4.91%ID/g and 33.00% ± 2.64%ID/g for Lf-PL[^99m^Tc] in comparison with 10.64% ± 0.77%ID/g and 10.91% ± 0.73%ID/g for PL[^99m^Tc], at 1 and 2 h post-injection, respectively. These results indicate that the Lf conjugated PL nanoparticles can significantly enhance the brain uptake in comparison with unmodified PL nanoparticles. Previous report revealed that the Lf receptor of mouse was localized in various regions of brain [21]. As such, the increase of the uptake in the brain for the Lf anchored liposomes may be mediated by the Lf receptors existed. In our biodistribution, spleen was the other organ shown significant difference in uptake levels. The marked increase of the splenic uptake of Lf-PL[^99m^Tc] over PL[^99m^Tc] might be attributed to the existing Lf ligands on the surface of Lf-PL liposomes which are easily bound by opsonins, subsequently recognized by RES system, and terminated in the spleen [14]. Lf receptor mediated function might be also recommended for the Lf-PL[^99m^Tc] increased uptake in the spleen but would not be confirmed with the scanty information of Lf receptors in the organ to date.

### 2.7. Prospective Development of Lf-PL

The related mechanism of Lf receptor in mammalian and/or in some brain diseases has been completely reviewed [22]. Most recently, Lf has been intensively studied for its brain-targeting capacity. A variety of nanoparticles including polyamidoamine dendrimer [7,23,24], poly(ethyleneglycol)-poly(lactide) [8], superparamagnetic iron oxide [9] and procationic liposome [15,16] had been utilized to conjugate with Lf as a vector and constructed as a promising gene or drug delivery system into the brain. In the present study, the PEGylated liposome nanoparticles (PL) was employed and Lf conjugated PL (Lf-PL) was investigated for its brain-targeting capacity. Liposomes are a common-used biocompatible nanoparticle, beneficial for its easily encapsulating hydrophilic or hydrophobic drugs into the internal water compartment or the membrane. We have shown the significant *in vitro* and *in vivo* results suggesting Lf-PL as a potential delivery system of the brain. Our radiotracer technique has also provided the biodistribution result in an animal model for the Lf conjugated nanoparticles for the first time. Lf receptor is existing abundantly not only on the cell surface of glioblastomas [9,10,16] but also in the lesion site of neurodegenerative diseases [12,13]. It would be worthy to develop Lf-PL[^99m^Tc] as a single photon emission computed tomography (SPECT) imaging agent for the diagnosis of glioblastomas and neurodegerative diseases or the similar product by ^188^Re, a ^99m^Tc surrogate, Lf-PL[^188^Re] as a radio-therapeutic agent for glioblastomas treatment.

## 3. Experimental Section

### 3.1. Materials

Distearoylphosphatidylcholine (DSPC), cholesterol, polyethylene glycol (MW~2 kDa)-distearoylphosphatidylethanolamine (DSPE-PEG_2000_), and 1,2-distearoyl-*sn*-glycero-3-phosphoethanolamine-*N*-[(polyethylene glycol) (MW ~3.4 kDa) maleimide] (DSPE-PEG_3400_-MAL) were all supplied from Genzyme (Cambridge, MA, USA). Traut’s Reagent (2-Iminothiolane·HCl) and Ellman’s Reagent were obtained from Thermo Scientific (Pierce Biotechnology, Rockford, IL, USA). Lf from bovine colostrum and Lf ELISA Quantitation Set were obtained from Sigma-Aldrich (St. Louis, MO, USA). *N*,*N*-bis(2-mercaptoethyl)-*N*′,*N*′-diethylethylenediamine (BMEDA) was from ABX (Radeberg, Germany). All other chemicals were from Merck (Darmstadt, Germany). PD-10 column and Sepharose 4 Fast Flow were from GE Healthcare (Uppsala, Sweden). Cell culture and media were from GIBCO BRL (Grand Island, NY, USA). ^99^Mo/^99m^Tc generator (TechneLite^®^) was supplied from Lantheus Medical Imaging, Inc. (N. Billerica, MA, USA).

### 3.2. Preparation of Maleimide Functional PEGylated Liposome (PL)

Maleimide (MAL) functional PL was prepared by the lipid film hydration-extrusion method using repeated freeze-thawing as described previously by Huang *et al.* [25], but with some modifications. Briefly, the mixture of DSPC: cholesterol: DSPE-PEG_2000_: DSPE-PEG_3400_-MAL at the molar ratio of 3:2:0.24:0.06 was dissolved in chloroform followed by removing the solvent by rotary evaporation. The resultant dry lipid film was rehydrated in 250 mM ammonium sulfate (pH 5.0) at 60 °C. After rehydration, the PEGylated liposomes was extruded 3 times through polycarbonate membrane filters with graded pore sizes (0.4, 0.2, 0.1, 0.05, and 0.03 μm) (Costar, Cambridge, MA, USA) via a high-pressure extruder (LIPEX™, Northern Lipids Inc., Burnaby, BC, Canada). Then the extraliposomal buffer was changed to normal saline via elution through a Sephadex G-50 column (Pharmacia, Uppsala, Sweden). The size and zeta potential of the nanoparticles were measured by a dynamic laser scattering (DLS) analyzer (N4 plus; Beckman Coulter). Phospholipid concentration was measured via phosphorus assay with UV-VIS spectrophotometry at λ = 830 nm (JascoV-530, Tokyo, Japan) [26].

### 3.3. Preparation of Lactoferrin Modified PEGylated Liposome (Lf-PL)

**Scheme 1:**
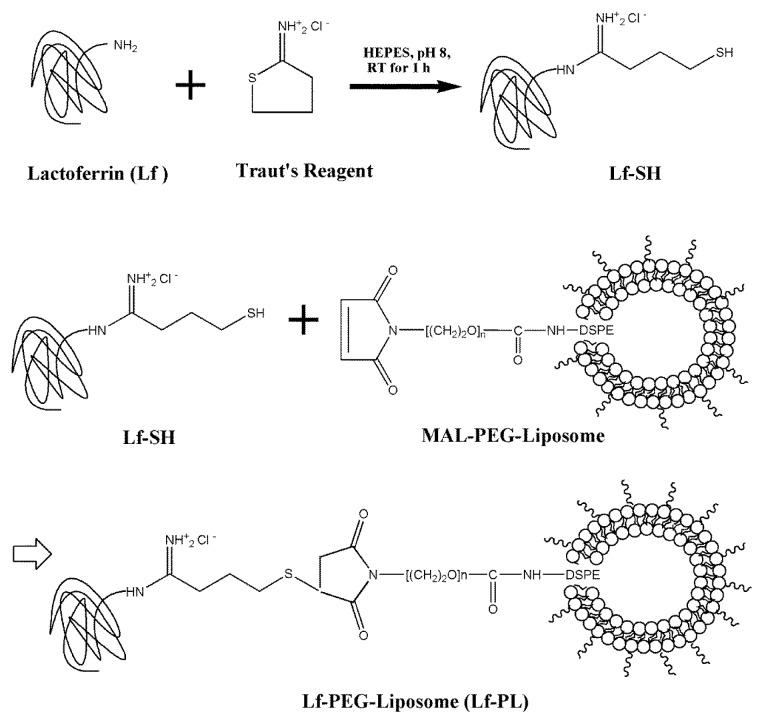
Fabrication of Lf-PL.

The procedure for fabrication of Lf-PL is shown in Scheme 1. In experiment, Lf was thiolated and conjugated to the distal MAL functional groups surrounding on PEGylated liposomes to form the product. Lf was derivated with a terminal sulfhydryl group at the *N*-termus by adding Traut’s Reagent as follows [27]. Traut’s Reagent (14.5 mM, 75 μL) and Lf (0.4 mg, 33.2 μL) were added together and dissolved in 20 mM HEPES (150 mM MgCl_2_, 2 mM EDTA, pH 8.0). The mixture was incubated at room temperature for 1 h and then passed through a PD-10 size exclusion column for purification. The resulted thiolated lactoferrin was added with 0.5 mL of PL at pH 6.8 at a molar ratio of 2:1 and reacted at room temperature for 17 h to form a thioether bonding with the MAL functional group at the *N*-terminus of DSPE-PEG_3400_-MAL chain on PL. l-cysteine (1 mg, 100 μL) was added and reacted for 30 min to block the unreacted MAL functional group. *N*-ethylmaleimide (8 mM) was then added to stop the above reaction. The resultant Lf-PL was purified via a Sepharose™ 4B column using normal saline as the eluent. The size and zeta potential of Lf-PL were measured via DLS analysis. The phospholipid concentration of Lf-PL was measured by phosphorus assay as precedingly described. The content of the terminal sulfhydryl group on Lf was determined by Ellman’s assay. The content of Lf in Lf-PL was measured by Bradford assay. The bioactivity of Lf for Lf-PL was validated by Lf ELISA assay. The number of Lf molecules conjugated on each liposome particle was further calculated based on the assumption of a 100 nm-liposome particle containing about 100,000 molecules of phospholipids [28].

### 3.4. Preparation of ^99m^Tc-BMEDA Complex, PL[^99m^Tc], and Lf-PL[^99m^Tc]

^99m^Tc in a form of NaTcO_4_ was obtained from a ^99^Mo/^99m^Tc generator by elution with normal saline. Labeling BMEDA with ^99m^Tc was carried out by the procedure reported by Bao *et al.* [29] and Chen *et al.* [30], but with some modifications. Briefly, BMEDA (5 mg) was pipetted into a vial. Then, 0.5 mL of 0.17 M sodium gluconate, 0.5 mL of 0.17 M acetate solution, and 120 μL of stannous chloride (10 mg/mL) were added. After flushing the solution with N^2^ gas, 1.5–3.7 GBq of Na^99m^TcO_4_ in saline was added. The mixture was heated at 80 °C for 1 h. The radiochemical yield for ^99m^Tc-BMEDA was measured by ITLC using silica gel impregnated glass fiber sheet (ITLC SG). The ITLC sheet was sectioned into eight pieces and the radioactivities were measured on an auto gamma counter (2480 WIZARD2^TM^, PerKinElmer, Waltham, MA, USA).

For preparation of PL[^99m^Tc] and Lf-PL[^99m^Tc], the ^99m^Tc-BMEDA solution was adjusted to pH 7.0 beforehand. The ^99m^Tc-BMEDA solution (0.74–1.85 GBq) was added to PL or Lf-PL (1 mL) and heated at 60 °C for 30 min. The ^99m^Tc loaded product, PL[^99m^Tc] or Lf-PL[^99m^Tc] was separated from the unreacted free ^99m^Tc-BMEDA using a PD-10 column eluted with normal saline. Into each tube was 0.5 mL fraction of the eluent collected. The fractions of the domain of PL[^99m^Tc] or Lf-PL[^99m^Tc] were visually monitored from the liposomal opacity. The radioactivties were measured on a radioisotope calibrator (CRC-15R). The loaded efficiency of PL[^99m^Tc] or Lf-PL[^99m^Tc] was calculated from the radioactivity in the domain of PL[^99m^Tc] or Lf-PL[^99m^Tc] divided by the total initial radioactivity added.

### 3.5. Stability Study

The stability of PL[^99m^Tc] as well as Lf-PL[^99m^Tc] during incubation in normal saline at room temperature and in rat plasma at 37 °C was studied. At each post-incubation time point (1, 4, 8, 20, 24, 30, and 48 h), a probe of 200 μL was taken and analyzed via a Poly-Prep chromography column (Bio-Red) packed with Sephadex G-50 or Sepharose 4B, by elution with normal saline. The radioactivities were measured on an auto gamma counter. The radiochemical purity was determined by the radioactivity of the separated product fractions divided by the total initial radioactivity of the sample loaded.

### 3.6. Cell Culture and Animals

bEnd.3 cells (BCRC 60515), the immortalized mouse brain endothelial cell line, were cultured in 90% Dulbecco’s modified Eagle’s medium supplemented with 4 mM l-glutamine, 1.5 g/L sodium bicarbonate, 4.5 g/L glucose and 10% fetal bovine serum, at 37 °C in a humidified environment with 5% CO_2_.

Normal male BALB/c mice (4~5-week old) were supplied from National Laboratory Animal Center (NLAC), Taiwan. All animal studies were approved by the Institutional Animal Care and Use Committee of National Tsing Hua University.

### 3.7. *In Vitro* Cell Uptake Study for Evaluation of BBB Penetration Potential

bEnd.3 cells [31] are of an immortalized mouse brain endothelial cell line. The cells are characterized as a model of the BBB by their rapid growth, maintenance of BBB characteristics over repeated passages, formation of functional barriers and amenability to numerous molecular interventions [19].

In *in vitro* cell uptake studies, bEnd.3 cells were seeded at 10^7^ cells/100 μL in an eppendorf tube and incubated at 37 °C with ^99m^Tc-BMEDA (0.4 MBq, 100 μL), PL[^99m^Tc] (0.4 MBq, 100 μL, 4 μmole/mL phospholipid), and Lf-PL[^99m^Tc] (0.4 MBq, 100 μL, 4 μmole/mL phospholipid), respectively for 0.5, 1, and 2 h. At each incubation time points, cells were separated by centrifugation (1500 × *g*, 10 min). An aliquot of 100 μL of the supernatant was transferred to another eppendof tube. Then both of the tubes with the respective contents were measured for radioactivities on an auto gamma counter (supposed to be A and B, respectively). The cell uptake index was calculated from the fomula—(A − B)/(A + B) [32].

### 3.8. Pharmacokinetic Study

Six normal male BALB/c mice (4~5 week-old) were used for the pharmacokinetic study. Animals were divided into two groups with three mice in each group. Mice were intravenously administrated with either PL[^99m^Tc] (3.7 MBq/100 μL, 4 μmole/mL) or Lf-PL[^99m^Tc] (3.7 MBq/100 μL, 4 μmole/mL). Blood samples were collected at 0.25, 0.5, 1, 2, 6, 20, and 24 h post-injection by cardiac puncture each with volume of 20–50 μL by a 29 gage needle after temporary anesthesia by 2% isoflurane [30]. The concentrations of radioactivity in the blood were calculated and expressed as percentage of injection dose per milliliter (%ID/mL). Pharmacokinetic parameters were determined using the WinNonlin softwere (version 5.0.1) (Pharsight: Mountain View, CA, USA). Non-compartmental analysis model 201 (IV-Bolus Input) was used along with the log/linear trapezoidal rule. Pharmacokinetic parameters including elimination half-life (T_1/2_), mean residence time (MRT), maximum concentration (C_max_), total body clearance (Cl), and area under the curve (AUC) were determined.

### 3.9. Biodistribution Study

Twelve normal male BABL/c mice (4–5 week-old) were used for the biodistribution study. Animals were divided into two groups with six mice in each group. Mice were intravenously administrated with either PL[^99m^Tc] (3.7 MBq/100 μL, 4 μmole/mL) or Lf-PL[^99m^Tc] (3.7 MBq/100 μL, 4 μmole/mL). Three mice were sacrificed at 1 and 2 h post-injection at each time point. The organs of interest including brain, blood, skin, kidney, spleen, liver, lung, and heart were dissected, rinsed in saline, blotted dry, weighed and then measured for radioactivities on an auto gamma counter. Aliquots of the injections of PL[^99m^Tc] and Lf-PL[^99m^Tc] were collected beforehand as the standard initial injected doses. The biodistribution results were expressed as percentage of injection dose per gram of organ or tissue (%ID/g).

### 3.10. Statistical Analysis

Data were expressed as mean ± standard deviation (SD). The unpaired t test was used for group comparisons. Values of *p* < 0.05 were considered significant.

## 4. Conclusions

The lactoferrin (Lf) conjugated PEGylated liposome (PL) was constructed in this study as a novel brain delivery system with evaluation of its *in vitro* and *in vivo* delivery properties. To evaluate the brain delivery properties of the Lf-PL, ^99m^Tc was incorporated into it as a radiotracer. The uptake of Lf-PL[^99m^Tc] by bEnd.3 cells was significantly higher than that of Lf-unconjugated PL[^99m^Tc]. The uptake of Lf-PL[^99m^Tc] in the brain was higher than liposome without the Lf targeting ligand in the animal study. Via probing by the radionuclide (^99m^Tc) in place of a commonly used fluorescin, our study showed the comparable results to the Lf conjugated nanoparticles *in vitro*. With the higher lactoferrin receptor density in various diseases, the conjugation strategy with SPECT radionuclide, ^99m^Tc, demonstrated the potential of imaging probe development for the diagnosis of neurodegenerative diseases and gliobastoma in the future.

## Figures and Tables

**Table 1:** Estimation of pharmacokinetic parameters of PL[^99m^Tc] and Lf-PL[^99m^Tc] after intravenous injection in male BALB/c mice (mean ± SD, *n* = 3).

Parameter	Unit	PL[^99m^Tc]	Lf-PL[^99m^Tc]

		0–24 h	0–24 h
T_1/2_	h	18.06 ± 2.09	13.88 ± 2.52
MRT	h	25.23 ± 3.07	19.81 ± 4.12
C_max_	%ID/mL	55.10 ± 1.79	53.61 ± 0.55
Cl	mL/h	0.09 ± 0.01	0.11 ± 0.02
AUC_(0→24h)_	h × %ID/mL	659.72 ± 58.41	653.57 ± 40.84

**Table 2:** Biodistribution results of PL[^99m^Tc] and Lf-PL[^99m^Tc] in BALB/c mice at 1 and 2 h post-injection.

Organ/tissue	PL[^99m^Tc]		Lf-PL[^99m^Tc]	

	1 h	2 h	1 h	2 h
brain *	0.69 ± 0.06	0.60 ± 0.04	1.02 ± 0.06	0.81 ± 0.05
blood *	46.82 ± 1.72	47.31 ± 1.48	34.38 ± 2.87	38.30 ± 2.44
bone	1.67 ± 0.57	2.66 ± 0.35	1.41 ± 1.25	2.43 ± 0.41
kidney	8.63 ± 0.74	9.23 ± 0.26	7.56 ± 0.86	9.36 ± 0.55
spleen *	10.64 ± 0.77	10.91 ± 0.73	34.12 ± 4.91	33.00 ± 2.64
liver	12.60 ± 3.49	10.83 ± 2.80	9.04 ± 2.89	11.15 ± 1.69
lung	10.05 ± 2.49	10.76 ± 2.39	10.61 ± 2.32	8.51 ± 0.82
heart	3.56 ± 2.51	4.84 ± 0.48	3.82 ± 0.46	4.15 ± 0.36

Values are presented as %ID/g, (mean ± SD, *n* = 3),

* indicates the organs or tissues with significantly different levels of ^99m^Tc uptake (*p* < 0.05).
